# The impact of diabetes on chronic pain in different body regions among adults aged 50 and older: a cross-sectional analysis

**DOI:** 10.3389/fpubh.2025.1520735

**Published:** 2025-02-20

**Authors:** Min Ding, Anle Ding, Lijie Zhu, Xiaoyun Xie

**Affiliations:** ^1^Department of Interventional and Vascular Surgery, Shanghai Tenth People’s Hospital, Tongji University School of Medicine, Shanghai, China; ^2^Huainan First People's Hospital, Huainan, Anhui, China

**Keywords:** diabetes mellitus, chronic pain, aged, blood glucose control, cross-sectional analysis

## Abstract

**Objective:**

This study investigates the association between diabetes and chronic pain across various body regions in individuals aged 50 years and older, while assessing the influence of gender, hypertension status, age, and glycemic control on this relationship.

**Methods:**

Data from the 2015 China Health and Retirement Longitudinal Study (CHARLS) database included 10,315 participants, with 1,983 diabetic and 8,332 non-diabetic individuals. Logistic regression models assessed the relationship between diabetes and chronic pain, adjusting for confounders. Subgroup analyses were conducted based on gender, age, and hypertension status, and the risk of pain in diabetic patients with well-controlled glycemia was compared to that in non-diabetic individuals.

**Results:**

Diabetes significantly increased the risk of pain in multiple body regions (*p* < 0.05). Diabetes was associated with a higher risk of headaches in males [OR = 1.33 (1.05–1. 69), *p* = 0.02] and individuals aged 65 or older [OR = 1.28 (1.04–1.58), *p* = 0.02]. Among non-hypertensive individuals, diabetes was not associated with an increased risk of pain. In females, hypertensives, and individuals under 65, diabetes significantly increased pain across multiple regions (*p* < 0.05). Diabetic individuals with well-controlled glycemia still showed a higher risk of finger [OR = 1.34 (1.03–1.76), *p* = 0.03] and toe pain [OR = 1.44 (1.05–1.99), *p* = 0.03] compared to non-diabetics.

**Conclusion:**

Diabetes is linked to increased pain in multiple body regions, especially in females, hypertensives, and those under 65. Even with good glycemic control, diabetic individuals remain at higher risk for finger and toe pain.

## 1 Introduction

Diabetes has become a major global public health challenge (1). In 2021, 529 million people worldwide were diagnosed, placing a heavy burden on patients and healthcare systems (2). Approximately half of diabetes patients have neuropathy, of whom 30 to 40% experience neuropathic pain (3), and diabetic patients are also more likely to suffer from other forms of chronic pain (4), severely impairing their quality of life and increasing healthcare costs (5).

However, studies investigating the association between diabetes and pain incidence, particularly among older adults, remain limited. A retrospective cohort study in Taiwan found that patients aged 18–50 with type 2 diabetes had a higher 10-year cumulative incidence of musculoskeletal pain and more frequent medical visits compared to non-diabetic individuals (6). Similarly, a study in Saudi Arabia involving 1,003 participants revealed a significant association between diabetes and prediabetes with chronic pain symptoms (7). In contrast, a survey study in Finland revealed that pain in older adults was more strongly associated with depressive states and comorbidities than with diabetes itself (8). Similarly, a prospective cohort study in the United States indicated that physical pain in older adults was closely related to poorer mental health and physical functioning, but not to poor blood glucose control (9). Additionally, a hospital-based study in Hong Kong found that pain was not significantly related to diabetes self-management (10).

Despite these insights, critical research gaps persist. First, there is a lack of detailed analysis of pain across specific body regions, particularly in the older population, limiting the understanding of the differential impact of diabetes on pain localization. Second, most large-scale studies on diabetes-related pain have been conducted in economically developed regions, such as the United States (9) and Finland (8), with limited representation from low-and middle-income regions. This geographic bias restricts the generalizability of current findings, as socioeconomic status, healthcare accessibility, and cultural influences are likely to significantly affect the manifestation and management of diabetes-related pain. For example, individuals in regions with better healthcare access may report less severe pain due to timely interventions, while those in underserved areas might face greater pain severity due to delayed diagnosis and treatment (11, 12).

Furthermore, few studies have conducted in-depth analyses of variations in diabetes-related pain across different population subgroups. Diabetes affect pain characteristics differently based on gender, age, and the presence of comorbidities, highlighting the need for large-scale, stratified research to address these gaps. Such studies would provide personalized insights into the relationship between diabetes and pain, ultimately informing targeted pain management interventions.

Therefore, this study aims to determine the association between diabetes and pain in specific body regions among adults aged 50 and older in China, utilizing nationally representative survey data to assess the effects of diabetes across different subgroups.

## 2 Methods

### 2.1 Survey design

This cross-sectional study used self-reported pain and diabetes diagnosis data from the 2015 China Health and Retirement Longitudinal Study (CHARLS) database to evaluate the impact of diabetes on the prevalence of chronic pain across various body sites in a diverse population aged 50 years and older.

### 2.2 Database

The CHARLS is a large-scale, national longitudinal study that collects information on demographic characteristics, health status, lifestyle, disease history, and socioeconomic conditions of middle-aged and older adults in China. Initiated in 2011, CHARLS has conducted follow-up surveys in 2013, 2015, 2018, and 2020 (13). The study protocol was approved by the Biomedical Ethics Committee of Peking University, and written informed consent was obtained from all participants.

### 2.3 Data selection

**Figure 1:**
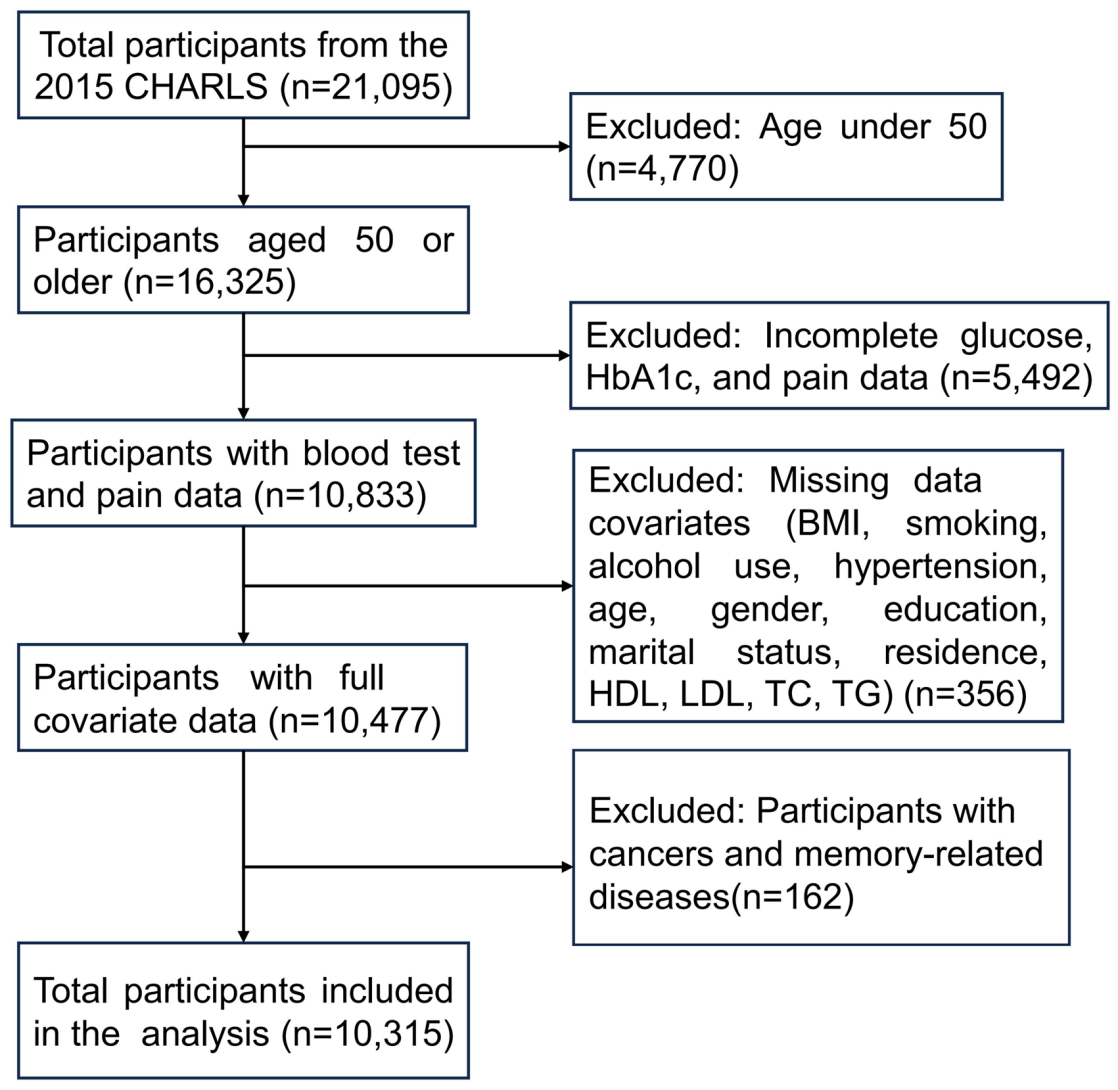
Flowchart of participants selection in this study.

We selected data from the 2015 CHARLS wave, focusing on participants primarily aged 50 and older, as this dataset provides the comprehensive covariates required for our analysis. Initially, data for 21,095 participants were obtained from the CHARLS database, with 16,325 individuals aged 50 or older. Pain-related data and blood test data relevant to diabetes, including glucose and glycated hemoglobin (HbA1c), were available for 10,833 individuals. Additionally, complete covariates data were accessible for 10,477 individuals, meeting the following criteria: non-missing values for body mass index (BMI), smoking status, alcohol consumption, hypertension, age, gender, education, marital status, residence, high-density lipoprotein (HDL), low-density lipoprotein (LDL), total cholesterol (TC), and triglycerides (TG). After excluding participants with cancers (except for minor skin cancers) and memory-related diseases, a total of 10,315 participants were ultimately included in the final analysis (Figure 1).

### 2.4 Definitions of diabetes and hypertension

Diabetes diagnosis was based on participants’ self-reported physician diagnoses or determined by the following criteria: (a) self-reported use of insulin or medications for diabetes treatment; (b) fasting plasma glucose (FPG) ≥ 7.0 mmol/L (126 mg/dL); (c) random blood glucose ≥11.1 mmol/L (200 mg/dL); (d) glycated hemoglobin (HbA1c) ≥ 6.5% (14).

Hypertension was defined as meeting any of the following criteria:(a) self-reported physician diagnosis of hypertension;(b) self-reported use of antihypertensive medication;(c) systolic blood pressure ≥ 140 mmHg or diastolic blood pressure ≥ 90 mmHg.

### 2.5 Pain assessment

Pain was assessed through self-reporting, with participants asked whether they experienced long-term chronic pain and specifying its location.

### 2.6 Blood glucose control

Glycemic control status was assessed through self-reported responses to the question: “Is your blood sugar generally under control?” Participants who answered “Yes” were categorized as having “Good Glycemic Control,” while those who answered “No” were classified as having “Poor Glycemic Control.”

### 2.7 Statistical analysis

Participants were classified based on their diabetes status. Descriptive statistical analyses summarized the baseline characteristics, including age, gender, BMI, smoking status, alcohol consumption, hypertension, education, marital status, residence, and lipid profiles (HDL, LDL, TC, and TG). Continuous variables following a normal distribution were expressed as means ± standard deviation (mean ± SD), with group differences evaluated using t test. Non-normally distributed continuous variables were presented as medians and interquartile ranges (IQR) and analyzed using the Mann–Whitney U test. Categorical variables were summarized as frequencies and percentages, and differences between groups were analyzed using the chi-square test. The Kolmogorov–Smirnov test was used to assess the normality of numerical distributions.

A univariate analysis was performed to investigate the association between diabetes and chronic pain across various body regions, using the chi-square test to compare pain incidence between diabetic and non-diabetic groups. Logistic regression models were used to evaluate the relationship between diabetes and chronic pain, with three models developed: Model 1 was unadjusted, Model 2 adjusted for age and gender, and Model 3 was fully adjusted for potential confounders including age, gender, BMI, education, marital status, hypertension, smoking, alcohol use, and lipid profiles (TC, TG, HDL, and LDL). Results are presented as odds ratios (OR) with 95% confidence intervals (CI). Stratified analyses were conducted to explore subgroup differences by gender, age, and hypertension status. Additionally, the impact of blood glucose control on chronic pain among diabetic individuals was analyzed.

All statistical analyses were performed using R software (version 4.2.3), and two-tailed *p*-values were reported. Statistical significance was set at *p* < 0.05.

## 3 Results

### 3.1 Sociodemographic and clinical characteristics

This study analyzed data from 10,315participants, including 1,983 individuals with diabetes and 8,332 without. Table 1 shows that the diabetes group was older, had a higher proportion of females, and a significantly greater percentage of urban residents compared to the non-diabetic group (*p* < 0.001). Additionally, the diabetes group exhibited higher BMI, and a significantly greater prevalence of hypertension (*p* < 0.001). Regarding biochemical indicators, HDL levels in the diabetes group were significantly lower than those in the non-diabetic group, while LDL, TC, and TG levels were significantly higher (*p* < 0.05). Supplementary Table 1 illustrates that the incidence of chronic pain in the diabetes group is significantly higher than that in the non-diabetic group, with an increased prevalence of pain in the head, shoulder, arm, fingers, chest, stomach, leg, and knees (*p* < 0.05).

**Table 1:** Baseline characteristics of participants stratified by diabetes status.

Variables	Total (*n* = 10,315)	Non-diabetic group (*n* = 8,332)	Diabetic group (*n* = 1983)	*p* value
Age, years, Median (IQR)	62.00 (56.00, 68.00)	62.00 (55.00, 68.00)	63.00 (58.00, 70.00)	<0.001
Age, group class, n (%)				<0.001
<65	6,313 (61.2%)	5,217 (62.6%)	1,096 (55.3%)	
≥65	4,002 (38.8%)	3,115 (37.4%)	887 (44.7%)	
Gender, n (%)				<0.001
Female	5,367 (52.0%)	4,274 (51.3%)	1,093 (55.1%)	
Male	4,948 (48.0%)	4,058 (48.7%)	890 (44.9%)	
Education, n (%)				0.9
Illiterate	809 (7.8%)	658 (7.9%)	151 (7.6%)	
Primary	1,027 (10.0%)	832 (10.0%)	195 (9.8%)	
Middle school	398 (3.9%)	322 (3.9%)	76 (3.8%)	
High school+	8,081 (78.3%)	6,520 (78.3%)	1,561 (78.7%)	
Marital, n (%)				0.055
Married	8,812 (85.4%)	7,142 (85.7%)	1,670 (84.2%)	
Single	1,503 (14.6%)	1,190 (14.3%)	313 (15.8%)	
Residence, n (%)				<0.001
Rural	8,635 (83.7%)	7,090 (85.1%)	1,545 (77.9%)	
Urban	1,680 (16.3%)	1,242 (14.9%)	438 (22.1%)	
BMI, group class, n (%)				<0.001
<18.5	622 (6.0%)	548 (6.6%)	74 (3.7%)	
18.5–23.9	5,005 (48.5%)	4,320 (51.8%)	685 (34.5%)	
24–27.9	3,382 (32.8%)	2,579 (31.0%)	803 (40.5%)	
≥28	1,306 (12.7%)	885 (10.6%)	421 (21.2%)	
Smoking, n (%)				<0.001
Non-smoker	5,687 (55.1%)	4,566 (54.8%)	1,121 (56.5%)	
Ex-smoker	1,429 (13.9%)	1,109 (13.3%)	320 (16.1%)	
Smoker	3,199 (31.0%)	2,657 (31.9%)	542 (27.3%)	
Alcohol use, n (%)				<0.001
YES	2,713 (26.3%)	2,265 (27.2%)	448 (22.6%)	
NO	7,602 (73.7%)	6,067 (72.8%)	1,535 (77.4%)	
Hypertension, n (%)				<0.001
YES	4,842 (46.9%)	3,595 (43.1%)	1,247 (62.9%)	
NO	5,473 (53.1%)	4,737 (56.9%)	736 (37.1%)	
HDL (mg/dl), Median (IQR)	49.81 (43.24, 57.53)	50.58 (43.63, 58.30)	47.10 (40.93, 54.05)	<0.001
LDL (mg/dl), Median (IQR)	101.54 (83.01, 120.08)	101.16 (83.01, 119.69)	103.86 (83.40, 123.55)	0.007
TC (mg/dl), Median (IQR)	182.24 (160.23, 206.56)	181.47 (159.46, 205.02)	186.87 (162.16, 213.13)	<0.001
TG (mg/dl), Median (IQR)	115.93 (83.19, 169.91)	110.62 (80.53, 161.06)	143.36 (99.12, 211.50)	<0.001

BMI, body mass index; HDL, High-Density Lipoprotein; LDL, Low-Density Lipoprotein; TC, Total cholesterol; TG, Triglycerides. Continuous variables, including age, HDL, LDL, TC, and TG, were determined to be non-normally distributed and were analyzed using the Mann–Whitney U test, whereas categorical variables were analyzed using the chi-square test.

### 3.2 Logistic regression analysis of diabetes and chronic pain

To assess the independent effects of diabetes on chronic pain, we developed three models using univariate and multivariate logistic regression (Table 2). Model 1 was unadjusted, Model 2 adjusted for age and gender, and Model 3 was fully adjusted for age, gender, BMI, education, marital status, hypertension, smoking, alcohol use, and lipid profiles (TC, TG, HDL, and LDL). The OR indicate the likelihood of experiencing pain, with CI representing the precision of these estimates. In Model 3, diabetes was significantly associated with a higher risk of pain across multiple body regions, including the head [OR = 1.26 (1.10–1.45), *p* < 0.001], arm [OR = 1.21 (1.04–1.41), *p* = 0.01], fingers [OR = 1.34 (1.14–1.59), *p* < 0.001], chest [OR = 1.25 (1.03–1.52), *p* = 0.02], stomach [OR = 1.24 (1.05–1.47), *p* = 0.01], and knees [OR = 1.16 (1.02–1.33), *p* = 0.03].

**Table 2:** Association between diabetes and chronic pain in different body regions based on univariate and multivariate logistic regression models.

	Model 1		Model 2		Model 3	
Location	OR (95%CI)	*p* value	OR (95%CI)	*p* value	OR (95%CI)	*p* value
Head	1.28 (1.12–1.46)	<0.001	1.25 (1.10–1.43)	<0.001	1.26 (1.10–1.45)	<0.001
Shoulder	1.17 (1.02–1.34)	0.02	1.13 (0.99–1.30)	0.07	1.14 (0.99–1.32)	0.06
Arm	1.24 (1.07–1.43)	0.003	1.2 (1.04–1.39)	0.01	1.21 (1.04–1.41)	0.01
Wrist	1.14 (0.96–1.35)	0.12	1.11 (0.94–1.32)	0.22	1.13 (0.95–1.35)	0.17
Fingers	1.38 (1.18–1.62)	<0.001	1.33 (1.14–1.57)	<0.001	1.34 (1.14–1.59)	<0.001
Chest	1.33 (1.10–1.60)	0.003	1.28 (1.07–1.55)	0.009	1.25 (1.03–1.52)	0.02
Stomach	1.21 (1.03–1.41)	0.02	1.19 (1.01–1.39)	0.03	1.24 (1.05–1.47)	0.01
Back	1.09 (0.94–1.27)	0.25	1.05 (0.90–1.22)	0.54	1.06 (0.91–1.24)	0.45
Waist	1.01 (0.90–1.14)	0.83	0.98 (0.87–1.11)	0.75	1.00 (0.88–1.13)	0.94
Buttocks	1.05 (0.86–1.28)	0.62	1.01 (0.82–1.23)	0.95	1.01 (0.82–1.25)	0.92
Leg	1.22 (1.08–1.39)	0.002	1.18 (1.04–1.34)	0.01	1.14 (1.00–1.30)	0.06
Knees	1.22 (1.07–1.38)	0.003	1.17 (1.03–1.33)	0.02	1.16 (1.02–1.33)	0.03
Ankle	1.15 (0.97–1.37)	0.11	1.11 (0.93–1.32)	0.27	1.07 (0.89–1.28)	0.49
Toes	1.2 (0.97–1.48)	0.09	1.12 (0.90–1.38)	0.31	1.11 (0.90–1.39)	0.33
Neck	1.01 (0.86–1.19)	0.91	0.98 (0.83–1.15)	0.79	0.96 (0.81–1.14)	0.65

Data were analyzed using univariate and multivariate logistic regression, with results expressed as odds ratios (OR) with 95% confidence intervals (CI) and p values. Non-diabetic participants served as the reference group for the analysis. Model 1: Unadjusted. Model 2: Adjusted for age and gender. Model 3: Adjusted for age, gender, BMI, education, marital status, hypertension, smoking, alcohol use, and lipid profiles (TC, TG, HDL, and LDL).

### 3.3 Subgroup analysis by sex, age, and hypertension status

Further subgroup analysis was conducted to explore whether the association between diabetes and chronic pain varied by sex, age, and hypertension status. In the sex-based analysis (Table 3), diabetes was significantly associated with an increased incidence of headaches only in men [OR = 1.33 (1.05–1.69), *p* = 0.02]. While in women, diabetes was linked to higher risks of pain in multiple regions, including head [OR = 1.24 (1.05–1.47), *p* = 0.01], arm [OR = 1.34 (1.11–1.61), *p* = 0.002], fingers [OR = 1.50 (1.23–1.82), *p* < 0.001], chest [OR = 1.32(1.05–1.67), *p* = 0.02], stomach [OR = 1.25 (1.02–1.53), *p* = 0.03], leg [OR = 1.24 (1.05–1.46), *p* = 0.01], knees [OR = 1.21 (1.03–1.43), *p* = 0.02], and toes [OR = 1.40 (1.09–1.79), *p* = 0.008].

**Table 3:** Association between diabetes and chronic pain across different body regions, stratified by gender.

	Male		Female		
Location	OR (95%CI)	*p* value	OR (95%CI)	*p* value	*P* for interaction
Head	1.33 (1.05–1.69)	0.02	1.24 (1.05–1.47)	0.01	0.632
Shoulder	1.13 (0.89–1.45)	0.32	1.15 (0.97–1.36)	0.12	0.794
Arm	1.02 (0.78–1.33)	0.89	1.34 (1.11–1.61)	0.002	0.079
Wrist	1.04 (0.75–1.45)	0.8	1.20 (0.97–1.48)	0.09	0.291
Fingers	1.06 (0.75–1.49)	0.75	1.50 (1.23–1.82)	<0.001	0.051
Chest	1.15 (0.82–1.62)	0.41	1.32 (1.05–1.67)	0.02	0.273
Stomach	1.25 (0.94–1.67)	0.13	1.25 (1.02–1.53)	0.03	0.998
Back	0.85 (0.62–1.15)	0.3	1.17 (0.97–1.41)	0.1	0.029
Waist	0.84 (0.67–1.05)	0.12	1.09 (0.93–1.27)	0.3	0.021
Buttocks	0.94 (0.63–1.40)	0.75	1.05 (0.82–1.34)	0.72	0.382
Leg	0.98 (0.77–1.25)	0.88	1.24 (1.05–1.46)	0.01	0.047
Knees	1.07 (0.84–1.35)	0.59	1.21 (1.03–1.43)	0.02	0.216
Ankle	0.85 (0.60–1.21)	0.37	1.19 (0.96–1.47)	0.12	0.074
Toes	0.54 (0.32–0.91)	0.02	1.40 (1.09–1.79)	0.008	0.001
Neck	0.85 (0.61–1.17)	0.31	1.02 (0.83–1.25)	0.84	0.332

Results are based on multivariate logistic regression models, adjusted for age, BMI, education, marital status, hypertension, smoking, alcohol use, and lipid profiles (TC, TG, HDL, and LDL). Non-diabetic participants served as the reference group for the analysis.

Age-based analysis (Table 4) revealed that among participants aged 65 and above, diabetes was primarily associated with an increased risk of headache [OR = 1.28 (1.04–1.58), *p* = 0.02]. In participants aged under 65, diabetes was significantly associated with higher risks of pain in multiple regions, including the head [OR = 1.24 (1.03–1.49), *p* = 0.02], shoulder [OR = 1.25 (1.04–1.51), *p* = 0.02], arm [OR = 1.27 (1.04–1.56), *p* = 0.02], fingers [OR = 1.40 (1.12–1.75), *p* = 0.003], stomach [OR = 1.32 (1.06–1.64), *p* = 0.01], and back [OR = 1.23 (1.00–1.51), *p* = 0.05].

**Table 4:** Association between diabetes and chronic pain across different body regions, stratified by age groups.

	<65 years		≥65 years		
Location	OR (95%CI)	*p* value	OR (95%CI)	*p* value	*P* for interaction
Head	1.24 (1.03–1.49)	0.02	1.28 (1.04–1.58)	0.02	0.767
Shoulder	1.25 (1.04–1.51)	0.02	1.00 (0.81–1.24)	1.00	0.097
Arm	1.27 (1.04–1.56)	0.02	1.14 (0.91–1.43)	0.26	0.446
Wrist	1.25 (0.99–1.57)	0.06	1.00 (0.76–1.31)	0.98	0.304
Fingers	1.40 (1.12–1.75)	0.003	1.27 (0.99–1.64)	0.06	0.572
Chest	1.29 (0.99–1.67)	0.06	1.20 (0.90–1.61)	0.20	0.807
Stomach	1.32 (1.06–1.64)	0.01	1.11 (0.86–1.44)	0.43	0.385
Back	1.23 (1.00–1.51)	0.05	0.89 (0.70–1.13)	0.33	0.024
Waist	1.02 (0.86–1.21)	0.78	0.94 (0.78–1.13)	0.51	0.714
Buttocks	1.12 (0.85–1.48)	0.42	0.89 (0.65–1.21)	0.45	0.325
Leg	1.08 (0.89–1.30)	0.44	1.21 (0.99–1.48)	0.06	0.327
Knees	1.14 (0.95–1.37)	0.17	1.17 (0.96–1.43)	0.11	0.781
Ankle	0.97 (0.75–1.25)	0.81	1.18 (0.91–1.54)	0.20	0.307
Toes	1.30 (0.97–1.76)	0.08	0.97 (0.71–1.33)	0.85	0.122
Neck	1.07 (0.86–1.34)	0.55	0.82 (0.63–1.07)	0.14	0.09

Results are based on multivariate logistic regression models, adjusted for gender, BMI, education, marital status, hypertension, smoking, alcohol use, and lipid profiles (TC, TG, HDL, and LDL). Non-diabetic participants served as the reference group for the analysis.

In the hypertension-based analysis (Supplementary Table 2), diabetes was not significantly increase the occurrence of pain in participants without hypertension. Among hypertensive participants, diabetes was associated with an increased risk of pain in multiple regions, including head [OR = 1.32 (1.11–1.57), *p* = 0.002], shoulder [OR = 1.25 (1.05–1.49), *p* = 0.01], arm [OR = 1.40 (1.16–1.69), *p* = 0.001], wrist [OR = 1.28 (1.02–1.60), *p* = 0.03], fingers [OR = 1.53 (1.23–1.89), *p* < 0.001], chest [OR = 1.38 (1.09–1.75), *p* = 0.008], stomach [OR = 1.36 (1.10–1.68), *p* = 0.005], leg [OR = 1.25 (1.05–1.47), *p* = 0.01], and knees [OR = 1.38 (1.16–1.63), *p* < 0.001].

### 3.4 Glycemic control and chronic pain prevalence in diabetes

We further analyzed the effect of blood glucose control on chronic pain in individuals with diabetes. Supplementary Tables 3, 4 indicate that diabetic individuals with good glycemic control had a lower incidence of generalized chronic pain, except in the ankle and toes, compared to those with poor glycemic control (*p* < 0.05). Table 5 compares diabetic individuals with good glycemic control to non-diabetic individuals, showing that those with well-controlled diabetes still exhibited an increased risk of pain in the fingers [OR = 1.34 (1.03–1.76), *p* = 0.03] and toes [OR = 1.44 (1.05–1.99), *p* = 0.03].

**Table 5:** Impact of diabetes on chronic pain in non-diabetic and good glycemic control diabetic individuals.

Location	OR (95%CI)	*p* value
Head	1.11 (0.88–1.40)	0.36
Shoulder	1.15 (0.91–1.44)	0.23
Arm	1.23 (0.97–1.57)	0.09
Wrist	1.09 (0.81–1.45)	0.58
Fingers	1.34 (1.03–1.76)	0.03
Chest	1.16 (0.85–1.59)	0.36
Stomach	1.08 (0.82–1.43)	0.59
Back	0.98 (0.75–1.27)	0.88
Waist	1.04 (0.85–1.28)	0.69
Buttocks	0.91 (0.64–1.28)	0.58
Leg	1.07 (0.86–1.33)	0.56
Knees	1.07 (0.86–1.34)	0.53
Ankle	0.99 (0.73–1.34)	0.96
Toes	1.44 (1.05–1.99)	0.03
Neck	0.93 (0.70–1.22)	0.59

Results are based on multivariate logistic regression models, adjusted for age, gender, BMI, education, marital status, hypertension, smoking, alcohol use, and lipid profiles (TC, TG, HDL, and LDL). Non-diabetic participants served as the reference group for the analysis.

## 4 Discussion

The findings of this study indicate that diabetes is significantly associated with an increased incidence of chronic pain in various body regions among individuals aged 50 and above, including the head, limbs, chest, and stomach. Subgroup analysis revealed that in males and individuals aged 65 and above, diabetes was associated solely with an increased risk of headaches. Among non-hypertensive individuals, diabetes was not linked to a higher risk of pain. In contrast, among females, hypertensive individuals, and those under 65 years of age, diabetes significantly increased the risk of chronic pain across multiple body regions. Effective blood glucose control can significantly reduce the incidence of pain in various body regions among diabetic individuals, with the exception of toe pain. However, diabetic individuals with well-controlled blood glucose levels still exhibited a higher risk of finger and toe pain compared to non-diabetic individuals.

The association between diabetes and pain locations identified in this study aligns with previous research findings. For example, a systematic review and meta-analysis, including six case–control studies and two cohort studies, demonstrated that diabetes is a risk factor for frozen shoulder (15). Previous research has shown that diabetic patients not only have a higher incidence of osteoarthritis (OA) (16), but also a risk factor for the progression of knee OA in older adults. A study of 559 individuals over 50 years old with symptomatic knee OA found that diabetes increases the risk of joint space narrowing in these patients (17). Beyond joint pain, neuropathic complications are of particular concern. Research indicates that approximately half of diabetes patients have neuropathy, of whom 30 to 40% accompanied by neuropathic pain, often manifesting as numbness and discomfort in a “glove and stocking” pattern (3). Additionally, diabetic patients face an increased risk of stomach pain, as diabetic autonomic neuropathy raises the likelihood of gastroparesis (18, 19), and certain antidiabetic medications may further exacerbate gastrointestinal discomfort (20). Overall, diabetes is associated with a heightened risk of chronic pain across multiple body regions.

Subgroup analysis revealed that diabetes had a greater impact on chronic pain in women and individuals with hypertension, consistent with previous findings. Research has shown that diabetic women face higher cardiovascular risks (21), which may be linked to chest and back pain. Additionally, diabetes increases the risk of osteoporosis (22) and sarcopenia (23) in postmenopausal women, elevating the incidence of musculoskeletal pain. Hypertension combined with diabetes leads to more severe cardiovascular damage (24), further raising the likelihood of pain in the chest (25) and limbs (26). An unexpected result in our study was the greater impact of diabetes on pain in individuals aged under 65 compared to individuals aged 65 and older. However, this finding is consistent with a Finnish community-based study, which surveyed 1,084 adults aged 65 or older and found no significant association between diabetes and pain intensity or pain interference. Instead, depressive symptoms and the number of comorbidities were linked to higher pain frequency (8). This suggests that in older patients, pain may be more complex and influenced by multiple other factors, reducing the relative impact of diabetes on pain. These subgroup analyses have important clinical implications. Healthcare providers should be alert to the heightened risk of chronic pain in diabetic patients, particularly women, those with hypertension, and individuals under 65. Tailored pain management strategies are crucial for these groups.

An interesting finding of this study is that diabetes significantly increased the incidence of headaches almost across all subgroups, regardless of gender and age status. Previous studies on the relationship between diabetes and headaches have produced conflicting results. Some research suggests a decreased prevalence of migraines in diabetic patients (27, 28), while others indicate an increased association (29–31), and some show no significant difference (32). These discrepancies may be due to differences in the study populations. Our study found that the overall prevalence of headaches was significantly higher in older adults with diabetes, but we did not differentiate between migraines and general headaches in our analysis.

The relationship between glycemic control and pain in diabetes, particularly in type 2 diabetes, has produced conflicting results in previous studies. A cohort study of type 2 diabetes patients in Rio de Janeiro found that glycemic control indices could predict the development and progression of peripheral neuropathy, a major cause of pain (33). Conversely, a prospective cohort study from the United States reported that pain is more closely associated with poor mental health and physical function than with poor glycemic control (9). Previous research has often lacked detailed analyses of pain specific to certain body regions. In contrast, our study found that while glycemic control did not significantly impact toe pain, it substantially alleviated pain in other body regions. In addition, healthcare system differences may influence outcomes. In countries with well-developed healthcare conditions, people with diabetes can receive timely treatment despite poor glycemic control, whereas in low-and middle-income countries, a large number of people with diabetes remain undiagnosed or untreated, leading to worsened pain outcomes due to poor glycemic control (11, 12).

Moreover, our study revealed that even with well-controlled blood glucose levels, diabetic participants still faced an increased risk of experiencing pain in the fingers and toes. This may be due to the common presence of metabolic syndrome among diabetic participants, which can cause peripheral nerve damage even with good glycemic control (34, 35). Additionally, if there was a history of poor glycemic control, the damage caused by hyperglycemia might persist even after achieving good control, particularly when irreversible damage has already occurred in peripheral nerves and microvessels (36). These findings highlight the importance of early glycemic control and weight management in diabetes care.

### 4.1 Limitations and strengths

This study has several limitations. The cross-sectional design prevents establishing causal relationships between diabetes and chronic pain. Additionally, the data primarily relied on participant self-reports and lacked detailed pain ratings and frequency assessments, which may reduce the precision of pain severity and occurrence comparisons. Furthermore, the dataset lacked information on neuropathy, a key complication of diabetes that significantly contributes to pain, which limits the scope of our findings. The study’s strengths include the use of the CHARLS database, which encompasses data from 28 provinces across China, providing a high degree of representativeness and timeliness. The study employed rigorous data screening and robust statistical analysis, offering a comprehensive examination of the impact of diabetes on chronic pain across different populations and ensuring the reliability and sensitivity of the findings.

## 5 Conclusion

In summary, diabetes significantly increases the risk of chronic pain in multiple body regions among individuals aged 50 and older, with the effect being especially pronounced in females, hypertensive individuals, and those under 65. Effective glycemic control can reduce the risk of chronic pain, but diabetic individuals remain at a higher risk of finger and toe pain compared to non-diabetic individuals.

## Data Availability

Publicly available datasets were analyzed in this study. This data can be found at: https://charls.charlsdata.com/.
