# Supplementary material for: The impact of diabetes on chronic pain in different body regions among adults aged 50 and older: a cross-sectional analysis
Source: Front Public Health. 2025 Feb 20;13:1520735. doi: 10.3389/fpubh.2025.1520735 (PMC11882414; doi:10.3389/fpubh.2025.1520735)
Supplement: Supplementary file 1 [file Table_1.docx]

Supplementary Material

# Supplementary Figures and Tables

**Supplementary Table 1** Incidence of Pain in Various Body Regions Among Diabetic and Non-Diabetic Individuals

| Location | Total  (n = 10315) | Non-Diabetic Group  (n = 8332) | Diabetic Group  (n = 1983) | *P* value |
| --- | --- | --- | --- | --- |
| Head, n (%) | 1534 (14.9%) | 1186 (14.2%) | 348 (17.5%) | <0.001 |
| Shoulder, n (%) | 1531 (14.8%) | 1204 (14.5%) | 327 (16.5%) | 0.022 |
| Arm, n (%) | 1227 (11.9%) | 953 (11.4%) | 274 (13.8%) | 0.003 |
| Wrist, n (%) | 893 (8.7%) | 704 (8.4%) | 189 (9.5%) | 0.124 |
| Fingers, n (%) | 931 (9.0%) | 706 (8.5%) | 225 (11.3%) | <0.001 |
| Chest, n (%) | 677 (6.6%) | 517 (6.2%) | 160 (8.1%) | 0.003 |
| Stomach, n (%) | 1006 (9.8%) | 785 (9.4%) | 221 (11.1%) | 0.02 |
| Back, n (%) | 1183 (11.5%) | 941 (11.3%) | 242 (12.2%) | 0.253 |
| Waist, n (%) | 2115 (20.5%) | 1705 (20.5%) | 410 (20.7%) | 0.833 |
| Buttocks, n (%) | 646 (6.3%) | 517 (6.2%) | 129 (6.5%) | 0.62 |
| Leg, n (%) | 1643 (15.9%) | 1282 (15.4%) | 361 (18.2%) | 0.002 |
| Knees, n (%) | 1688 (16.4%) | 1319 (15.8%) | 369 (18.6%) | 0.003 |
| Ankle, n (%) | 824 (8.0%) | 648 (7.8%) | 176 (8.9%) | 0.105 |
| Toes, n (%) | 545 (5.3%) | 425 (5.1%) | 120 (6.1%) | 0.089 |
| Neck, n (%) | 1023 (9.9%) | 825 (9.9%) | 198 (10.0%) | 0.911 |

**Supplementary Table 2** Association Between Diabetes and Chronic Pain Across Body Regions in Hypertensive and Non-Hypertensive Subgroups

|  | Non-Hypertensive | | Hypertensive | |  |
| --- | --- | --- | --- | --- | --- |
| Location | OR (95%CI) | *P* value | OR (95%CI) | *P* value | *P* for interaction |
| Head | 1.19 (0.95–1.50) | 0.13 | 1.32 (1.11–1.57) | 0.002 | 0.494 |
| Shoulder | 0.99 (0.79–1.25) | 0.93 | 1.25 (1.05–1.49) | 0.01 | 0.189 |
| Arm | 0.97 (0.75–1.25) | 0.81 | 1.40 (1.16–1.69) | 0.001 | 0.033 |
| Wrist | 0.94 (0.70–1.26) | 0.69 | 1.28 (1.02–1.60) | 0.03 | 0.166 |
| Fingers | 1.11 (0.84–1.47) | 0.44 | 1.53 (1.23–1.89) | <0.001 | 0.161 |
| Chest | 1.03 (0.74–1.45) | 0.85 | 1.38 (1.09–1.75) | 0.008 | 0.179 |
| Stomach | 1.10 (0.84–1.43) | 0.51 | 1.36 (1.10–1.68) | 0.005 | 0.278 |
| Back | 0.94 (0.72–1.22) | 0.62 | 1.15 (0.94–1.40) | 0.18 | 0.249 |
| Waist | 0.77 (0.63–0.96) | 0.02 | 1.16 (0.99–1.36) | 0.07 | 0.005 |
| Buttocks | 0.96 (0.68–1.36) | 0.83 | 1.05 (0.80–1.36) | 0.74 | 0.605 |
| Leg | 0.97 (0.77–1.22) | 0.81 | 1.25 (1.05–1.47) | 0.01 | 0.079 |
| Knees | 0.86 (0.68–1.08) | 0.21 | 1.38 (1.16–1.63) | <0.001 | 0.002 |
| Ankle | 1.01 (0.75–1.37) | 0.93 | 1.10 (0.87–1.38) | 0.43 | 0.625 |
| Toes | 1.01 (0.69–1.47) | 0.96 | 1.18 (0.90–1.55) | 0.23 | 0.466 |
| Neck | 0.86 (0.65–1.15) | 0.31 | 1.04 (0.84–1.28) | 0.75 | 0.658 |

Results are based on multivariate logistic regression models, adjusted for age, gender, BMI, education, marital status, smoking, alcohol use, and lipid profiles (TC, TG, HDL, and LDL). Non-diabetic participants served as the reference group for the analysis.

**Supplementary Table 3** Incidence of Pain in Various Body Regions Among Diabetic Individuals with Good and Poor Glycemic Control

| Location | Total(n = 918) | Poor Glycemic Control (n = 283) | Good Glycemic Control (n = 635) | *P* value |
| --- | --- | --- | --- | --- |
| Head, n (%) | 176 (19.2%) | 75 (26.5%) | 101 (15.9%) | <0.001 |
| Shoulder, n (%) | 186 (20.3%) | 81 (28.6%) | 105 (16.5%) | <0.001 |
| Arm, n (%) | 153 (16.7%) | 65 (23.0%) | 88 (13.9%) | <0.001 |
| Wrist, n (%) | 104 (11.3%) | 46 (16.3%) | 58 (9.1%) | 0.002 |
| Fingers, n (%) | 125 (13.6%) | 54 (19.1%) | 71 (11.2%) | 0.001 |
| Chest, n (%) | 87 (9.5%) | 39 (13.8%) | 48 (7.6%) | 0.003 |
| Stomach, n (%) | 109 (11.9%) | 47 (16.6%) | 62 (9.8%) | 0.003 |
| Back, n (%) | 133 (14.5%) | 61 (21.6%) | 72 (11.3%) | <0.001 |
| Waist, n (%) | 226 (24.6%) | 90 (31.8%) | 136 (21.4%) | <0.001 |
| Buttocks, n (%) | 66 (7.2%) | 28 (9.9%) | 38 (6.0%) | 0.034 |
| Leg, n (%) | 191 (20.8%) | 81 (28.6%) | 110 (17.3%) | <0.001 |
| Knees, n (%) | 196 (21.4%) | 85 (30.0%) | 111 (17.5%) | <0.001 |
| Ankle, n (%) | 87 (9.5%) | 34 (12.0%) | 53 (8.3%) | 0.08 |
| Toes, n (%) | 65 (7.1%) | 17 (6.0%) | 48 (7.6%) | 0.397 |
| Neck, n (%) | 114 (12.4%) | 52 (18.4%) | 62 (9.8%) | <0.001 |

The incidence of pain across different body regions among diabetic individuals with good and poor glycemic control was analyzed using the chi-square test.

**Supplementary Table 4** Association Between Glycemic Control and Chronic Pain in Different Body Regions Among Diabetic Individuals

| Location | OR (95%CI) | *P* value |
| --- | --- | --- |
| Head | 0.53 (0.37–0.76) | <0.001 |
| Shoulder | 0.49 (0.34–0.69) | <0.001 |
| Arm | 0.55 (0.38–0.80) | 0.002 |
| Wrist | 0.49 (0.32–0.75) | 0.001 |
| Fingers | 0.51 (0.34–0.77) | 0.001 |
| Chest | 0.54 (0.34–0.85) | 0.008 |
| Stomach | 0.57 (0.37–0.88) | 0.01 |
| Back | 0.45 (0.31–0.67) | <0.001 |
| Waist | 0.60 (0.43–0.83) | 0.002 |
| Buttocks | 0.60 (0.35–1.01) | 0.05 |
| Leg | 0.50 (0.35–0.71) | <0.001 |
| Knees | 0.49 (0.35–0.70) | <0.001 |
| Ankle | 0.63 (0.39–1.01) | 0.05 |
| Toes | 1.25 (0.69–2.26) | 0.47 |
| Neck | 0.47 (0.31–0.71) | <0.001 |

Results are based on multivariate logistic regression models, adjusted for age, gender, BMI, education, marital status, hypertension, smoking, alcohol use, and lipid profiles (TC, TG, HDL, and LDL). Poor Glycemic Control group served as the reference group for the analysis.

Result：Among the diabetic participants in this study, 918 individuals responded to the glycemic control questionnaire, with 283 reporting poor control and 635 reporting good control. We compared the prevalence of pain across these groups. Chi-square tests showed that patients with good glycemic control experienced significantly lower pain prevalence across all body regions, except for the ankle and toes(STable 3). Logistic regression analyses confirmed these findings (*p* < 0.05)(STable 4).
